# Supplementary material for: Influence of periodontal treatment on blood microbiotas: a clinical trial
Source: PeerJ. 2021 Feb 16;9:e10846. doi: 10.7717/peerj.10846 (PMC7894104; doi:10.7717/peerj.10846)
Supplement: Supplemental Information 9 [file peerj-09-10846-s009.doc]

报告单号:D2019120205

**奥维森基因扩增子样品质检报告**

1. 项目信息

| 合同编号 | AWGT19101811 | 项目名称 | 青岛市口腔医院86个血液DNA样本16SV3-V4区MiseqPE300测序分析ZWY |
| --- | --- | --- | --- |
| 合作单位 | 【张文怡】青岛市口腔医院 | 客户姓名 | 张文怡 |
| 样品接收人 | 戴璐敏 | 接收日期 | 2019-10-18 |
| 样品检测人 | 魏凌峰 | 检测日期 | 2019-10-29 |
| 报告撰写人 | 张洋 | 发布日期 | 2019-12-02 |
| 报告审核人 | 张洋 | | |

1. **样品检测方法**

Meta Amplicon DNA样品检测方法：PCR扩增。

1. 样品检测结果

| **序号** | **样品名称** | **样品编号** | **扩增区域** | **检测结果** | **检测等级** | **备注** |
| --- | --- | --- | --- | --- | --- | --- |
| **1** | **N5** | **SD20191018200** | **16S V3-V4** | **不合格** | **D** |  |
| **2** | **N55** | **SD20191018201** | **16S V3-V4** | **不合格** | **D** |  |
| **3** | **N52** | **SD20191018202** | **16S V3-V4** | **不合格** | **C** |  |
| **4** | **N45** | **SD20191018203** | **16S V3-V4** | **不合格** | **C** |  |
| **5** | **N44** | **SD20191018204** | **16S V3-V4** | **不合格** | **C** |  |
| **6** | **N51** | **SD20191018205** | **16S V3-V4** | **不合格** | **C** |  |
| **7** | **N24** | **SD20191018206** | **16S V3-V4** | **不合格** | **C** |  |
| **8** | **N67** | **SD20191018207** | **16S V3-V4** | **不合格** | **C** |  |
| **9** | **N46** | **SD20191018208** | **16S V3-V4** | **不合格** | **C** |  |
| **10** | **N1** | **SD20191018209** | **16S V3-V4** | **不合格** | **D** |  |
| **11** | **N28** | **SD20191018210** | **16S V3-V4** | **不合格** | **C** |  |
| **12** | **N14** | **SD20191018211** | **16S V3-V4** | **合格** | **B** |  |
| **13** | **N6** | **SD20191018212** | **16S V3-V4** | **合格** | **B** |  |
| **14** | **N61** | **SD20191018213** | **16S V3-V4** | **不合格** | **C** |  |
| **15** | **N48** | **SD20191018214** | **16S V3-V4** | **合格** | **B** |  |
| **16** | **N15** | **SD20191018215** | **16S V3-V4** | **合格** | **B** |  |
| **17** | **N59** | **SD20191018216** | **16S V3-V4** | **不合格** | **C** |  |
| **18** | **N26** | **SD20191018217** | **16S V3-V4** | **不合格** | **C** |  |
| **19** | **Q43** | **SD20191018218** | **16S V3-V4** | **不合格** | **C** |  |
| **20** | **Q43B** | **SD20191018219** | **16S V3-V4** | **不合格** | **C** |  |
| **21** | **Q43c** | **SD20191018220** | **16S V3-V4** | **不合格** | **C** |  |
| **22** | **ZX** | **SD20191018221** | **16S V3-V4** | **不合格** | **C** |  |
| **23** | **Q40B** | **SD20191018222** | **16S V3-V4** | **不合格** | **C** |  |
| **24** | **Q40C** | **SD20191018223** | **16S V3-V4** | **不合格** | **C** |  |
| **25** | **Q57** | **SD20191018224** | **16S V3-V4** | **不合格** | **C** |  |
| **26** | **Q57B** | **SD20191018225** | **16S V3-V4** | **合格** | **B** |  |
| **27** | **Q57C** | **SD20191018226** | **16S V3-V4** | **不合格** | **C** |  |
| **28** | **Q61** | **SD20191018227** | **16S V3-V4** | **不合格** | **C** |  |
| **29** | **Q61B** | **SD20191018228** | **16S V3-V4** | **合格** | **B** |  |
| **30** | **Q61C** | **SD20191018229** | **16S V3-V4** | **不合格** | **C** |  |
| **31** | **Q55** | **SD20191018230** | **16S V3-V4** | **不合格** | **C** |  |
| **32** | **Q55B** | **SD20191018231** | **16S V3-V4** | **不合格** | **D** |  |
| **33** | **Q55c** | **SD20191018232** | **16S V3-V4** | **不合格** | **C** |  |
| **34** | **Q69** | **SD20191018233** | **16S V3-V4** | **不合格** | **C** |  |
| **35** | **Q69B** | **SD20191018234** | **16S V3-V4** | **不合格** | **C** |  |
| **36** | **Q25** | **SD20191018235** | **16S V3-V4** | **不合格** | **C** |  |
| **37** | **Q25B** | **SD20191018236** | **16S V3-V4** | **合格** | **B** |  |
| **38** | **Q25C** | **SD20191018237** | **16S V3-V4** | **合格** | **B** |  |
| **39** | **Q28** | **SD20191018238** | **16S V3-V4** | **合格** | **B** |  |
| **40** | **Q28B** | **SD20191018239** | **16S V3-V4** | **合格** | **B** |  |
| **41** | **Q28C** | **SD20191018240** | **16S V3-V4** | **不合格** | **C** |  |
| **42** | **JLH** | **SD20191018241** | **16S V3-V4** | **不合格** | **C** |  |
| **43** | **Q33B** | **SD20191018242** | **16S V3-V4** | **合格** | **A** |  |
| **44** | **Q33C** | **SD20191018243** | **16S V3-V4** | **合格** | **B** |  |
| **45** | **Q38** | **SD20191018244** | **16S V3-V4** | **不合格** | **C** |  |
| **46** | **Q38B** | **SD20191018245** | **16S V3-V4** | **不合格** | **C** |  |
| **47** | **Q50** | **SD20191018246** | **16S V3-V4** | **不合格** | **C** |  |
| **48** | **Q50B** | **SD20191018247** | **16S V3-V4** | **不合格** | **C** |  |
| **49** | **Q50C** | **SD20191018248** | **16S V3-V4** | **合格** | **B** |  |
| **50** | **Q23** | **SD20191018249** | **16S V3-V4** | **合格** | **B** |  |
| **51** | **QL2** | **SD20191018250** | **16S V3-V4** | **合格** | **B** |  |
| **52** | **Q23C** | **SD20191018251** | **16S V3-V4** | **不合格** | **D** |  |
| **53** | **Q26** | **SD20191018252** | **16S V3-V4** | **合格** | **B** |  |
| **54** | **Q26B** | **SD20191018253** | **16S V3-V4** | **不合格** | **C** |  |
| **55** | **Q26C** | **SD20191018254** | **16S V3-V4** | **合格** | **A** |  |
| **56** | **Q34** | **SD20191018255** | **16S V3-V4** | **合格** | **A** |  |
| **57** | **Q34B** | **SD20191018256** | **16S V3-V4** | **不合格** | **C** |  |
| **58** | **Q34C** | **SD20191018257** | **16S V3-V4** | **合格** | **B** |  |
| **59** | **Q35** | **SD20191018258** | **16S V3-V4** | **不合格** | **C** |  |
| **60** | **Q35B** | **SD20191018259** | **16S V3-V4** | **不合格** | **C** |  |
| **61** | **Q35C** | **SD20191018260** | **16S V3-V4** | **不合格** | **C** |  |
| **62** | **Q36** | **SD20191018261** | **16S V3-V4** | **合格** | **B** |  |
| **63** | **Q36B** | **SD20191018262** | **16S V3-V4** | **不合格** | **C** |  |
| **64** | **Q36C** | **SD20191018263** | **16S V3-V4** | **不合格** | **C** |  |
| **65** | **Q37** | **SD20191018264** | **16S V3-V4** | **不合格** | **A** |  |
| **66** | **Q37B** | **SD20191018265** | **16S V3-V4** | **合格** | **B** |  |
| **67** | **Q54** | **SD20191018266** | **16S V3-V4** | **合格** | **B** |  |
| **68** | **Q54B** | **SD20191018267** | **16S V3-V4** | **合格** | **B** |  |
| **69** | **Q54C** | **SD20191018268** | **16S V3-V4** | **合格** | **B** |  |
| **70** | **Q58** | **SD20191018269** | **16S V3-V4** | **不合格** | **C** |  |
| **71** | **Q58B** | **SD20191018270** | **16S V3-V4** | **合格** | **A** |  |
| **72** | **Q47** | **SD20191018271** | **16S V3-V4** | **不合格** | **D** |  |
| **73** | **QB** | **SD20191018272** | **16S V3-V4** | **不合格** | **C** |  |
| **74** | **Q24** | **SD20191018273** | **16S V3-V4** | **不合格** | **C** |  |
| **75** | **Q24B** | **SD20191018274** | **16S V3-V4** | **不合格** | **D** |  |
| **76** | **Q24C** | **SD20191018275** | **16S V3-V4** | **不合格** | **D** |  |
| **77** | **Q27** | **SD20191018276** | **16S V3-V4** | **不合格** | **C** |  |
| **78** | **Q27B** | **SD20191018277** | **16S V3-V4** | **不合格** | **C** |  |
| **79** | **Q27C** | **SD20191018278** | **16S V3-V4** | **不合格** | **D** |  |
| **80** | **Q19** | **SD20191018279** | **16S V3-V4** | **不合格** | **C** |  |
| **81** | **Q19B** | **SD20191018280** | **16S V3-V4** | **合格** | **B** |  |
| **82** | **Q19C** | **SD20191018281** | **16S V3-V4** | **不合格** | **D** |  |
| **83** | **Q18B** | **SD20191018282** | **16S V3-V4** | **不合格** | **D** |  |
| **84** | **Q18C** | **SD20191018283** | **16S V3-V4** | **不合格** | **D** |  |
| **85** | **Q21** | **SD20191018284** | **16S V3-V4** | **不合格** | **C** |  |
| **86** | **Q21B** | **SD20191018285** | **16S V3-V4** | **不合格** | **D** |  |

***注：**

1. 检测结果和备注是依据DNA样品判定标准，对被检测样品是否符合要求给出的意见和解释，检测结果类别分类要求如下：
2. PCR产物目的条带大小正确，且总量满足2次或者2次以上建库需要；
3. PCR产物目的条带大小正确，且总量满足1次但不足2次建库需要；
4. PCR产物有非特异性条带，目的条带浓度低，可风险建库但不保证测序质量；
5. PCR产物无目的条带或浓度低不能满足建库要求，不建议使用，需重新送样。

**四、附件**

1. 检测前处理

将样品在冰上融化后，离心并充分混匀，Nanodrop检测样品质量，取30ng进行PCR扩增。

1. PCR扩增体系

| 试剂成分 | 体积 |
| --- | --- |
| DNA样品 | X (30ng) |
| Forward Primer(5 uM) | 1μL |
| Reverse Primer(5uM) | 1μL |
| BSA(2 ng/μL) | 3 μL |
| 2xTaq Plus Master Mix | 12.5 μL |
| ddH_2_O | 7.5-X μL |
| Total | 25μL |

1. 扩增引物序列

第一轮：

| 扩增区域 | 引物序列 |
| --- | --- |
| 16S V3-V4 | AGAGTTTGATCMTGGCTCAG |
|  | TACGGYTACCTTGTTACGACTT |

第二轮:

| 扩增区域 | 引物序列 |
| --- | --- |
| 16S V3-V4 | GTACTCCTACGGGAGGCAGCA |
|  | GTGGACTACHVGGGTWTCTAAT |

1. PCR扩增程序

第一轮:

| 94℃ 5 min |  |
| --- | --- |
| 94℃ 60 s | 30 Cycles |
| 55℃ 30 s |
| 72℃ 120s |
| 72℃ 7 min |  |
| 4℃ end |  |

第二轮：

| 94℃ 5 min |  |
| --- | --- |
| 94℃ 30 s | 30 Cycles |
| 50℃ 30 s |
| 72℃ 60s |
| 72℃ 7 min |  |
| 4℃ end |  |

1. 琼脂糖凝胶电泳检测参数

胶浓度：1%；电压：170V; 电泳时间：30min

1. 琼脂糖凝胶电泳检测结果


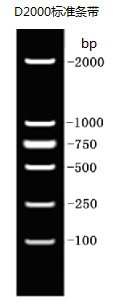


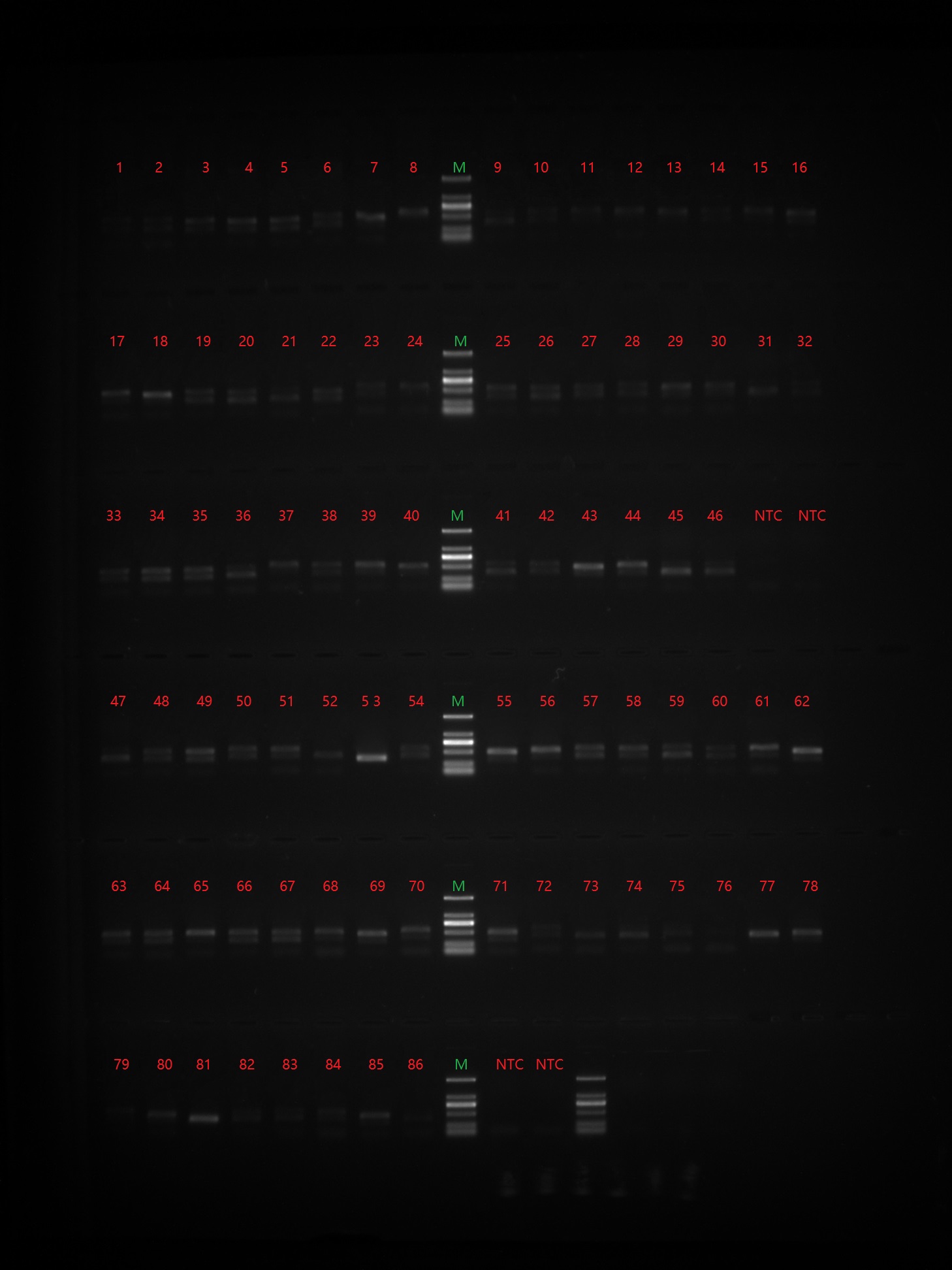


| **点孔**  **次数** | **样品名称** | **稀释倍数(×)** | **上样量(μL)** | **扩增区域** |
| --- | --- | --- | --- | --- |
| 1 | N5 | 1 | 3 | 16S V3-V4 |
| 2 | N55 | 1 | 3 | 16S V3-V4 |
| 3 | N52 | 1 | 3 | 16S V3-V4 |
| 4 | N45 | 1 | 3 | 16S V3-V4 |
| 5 | N44 | 1 | 3 | 16S V3-V4 |
| 6 | N51 | 1 | 3 | 16S V3-V4 |
| 7 | N24 | 1 | 3 | 16S V3-V4 |
| 8 | N67 | 1 | 3 | 16S V3-V4 |
| M | D2000 DNA Marker | 1 | 3 |  |
| 9 | N46 | 1 | 3 | 16S V3-V4 |
| 10 | N1 | 1 | 3 | 16S V3-V4 |
| 11 | N28 | 1 | 3 | 16S V3-V4 |
| 12 | N14 | 1 | 3 | 16S V3-V4 |
| 13 | N6 | 1 | 3 | 16S V3-V4 |
| 14 | N61 | 1 | 3 | 16S V3-V4 |
| 15 | N48 | 1 | 3 | 16S V3-V4 |
| 16 | N15 | 1 | 3 | 16S V3-V4 |
| 17 | N59 | 1 | 3 | 16S V3-V4 |
| 18 | N26 | 1 | 3 | 16S V3-V4 |
| 19 | Q43 | 1 | 3 | 16S V3-V4 |
| 20 | Q43B | 1 | 3 | 16S V3-V4 |
| 21 | Q43c | 1 | 3 | 16S V3-V4 |
| 22 | ZX | 1 | 3 | 16S V3-V4 |
| 23 | Q40B | 1 | 3 | 16S V3-V4 |
| 24 | Q40C | 1 | 3 | 16S V3-V4 |
| M | D2000 DNA Marker | 1 | 3 |  |
| 25 | Q57 | 1 | 3 | 16S V3-V4 |
| 26 | Q57B | 1 | 3 | 16S V3-V4 |
| 27 | Q57C | 1 | 3 | 16S V3-V4 |
| 28 | Q61 | 1 | 3 | 16S V3-V4 |
| 29 | Q61B | 1 | 3 | 16S V3-V4 |
| 30 | Q61C | 1 | 3 | 16S V3-V4 |
| 31 | Q55 | 1 | 3 | 16S V3-V4 |
| 32 | Q55B | 1 | 3 | 16S V3-V4 |
| 33 | Q55c | 1 | 3 | 16S V3-V4 |
| 34 | Q69 | 1 | 3 | 16S V3-V4 |
| 35 | Q69B | 1 | 3 | 16S V3-V4 |
| 36 | Q25 | 1 | 3 | 16S V3-V4 |
| 37 | Q25B | 1 | 3 | 16S V3-V4 |
| 38 | Q25C | 1 | 3 | 16S V3-V4 |
| 39 | Q28 | 1 | 3 | 16S V3-V4 |
| 40 | Q28B | 1 | 3 | 16S V3-V4 |
| M | D2000 DNA Marker | 1 | 3 |  |
| 41 | Q28C | 1 | 3 | 16S V3-V4 |
| 42 | JLH | 1 | 3 | 16S V3-V4 |
| 43 | Q33B | 1 | 3 | 16S V3-V4 |
| 44 | Q33C | 1 | 3 | 16S V3-V4 |
| 45 | Q38 | 1 | 3 | 16S V3-V4 |
| 46 | Q38B | 1 | 3 | 16S V3-V4 |
|  | NTC | 1 | 3 | 16S V3-V4 |
|  | NTC | 1 | 3 | 16S V3-V4 |
| 47 | Q50 | 1 | 3 | 16S V3-V4 |
| 48 | Q50B | 1 | 3 | 16S V3-V4 |
| 49 | Q50C | 1 | 3 | 16S V3-V4 |
| 50 | Q23 | 1 | 3 | 16S V3-V4 |
| 51 | QL2 | 1 | 3 | 16S V3-V4 |
| 52 | Q23C | 1 | 3 | 16S V3-V4 |
| 53 | Q26 | 1 | 3 | 16S V3-V4 |
| 54 | Q26B | 1 | 3 | 16S V3-V4 |
| M | D2000 DNA Marker | 1 | 3 |  |
| 55 | Q26C | 1 | 3 | 16S V3-V4 |
| 56 | Q34 | 1 | 3 | 16S V3-V4 |
| 57 | Q34B | 1 | 3 | 16S V3-V4 |
| 58 | Q34C | 1 | 3 | 16S V3-V4 |
| 59 | Q35 | 1 | 3 | 16S V3-V4 |
| 60 | Q35B | 1 | 3 | 16S V3-V4 |
| 61 | Q35C | 1 | 3 | 16S V3-V4 |
| 62 | Q36 | 1 | 3 | 16S V3-V4 |
| 63 | Q36B | 1 | 3 | 16S V3-V4 |
| 64 | Q36C | 1 | 3 | 16S V3-V4 |
| 65 | Q37 | 1 | 3 | 16S V3-V4 |
| 66 | Q37B | 1 | 3 | 16S V3-V4 |
| 67 | Q54 | 1 | 3 | 16S V3-V4 |
| 68 | Q54B | 1 | 3 | 16S V3-V4 |
| 69 | Q54C | 1 | 3 | 16S V3-V4 |
| 70 | Q58 | 1 | 3 | 16S V3-V4 |
| M | D2000 DNA Marker | 1 | 3 |  |
| 71 | Q58B | 1 | 3 | 16S V3-V4 |
| 72 | Q47 | 1 | 3 | 16S V3-V4 |
| 73 | QB | 1 | 3 | 16S V3-V4 |
| 74 | Q24 | 1 | 3 | 16S V3-V4 |
| 75 | Q24B | 1 | 3 | 16S V3-V4 |
| 76 | Q24C | 1 | 3 | 16S V3-V4 |
| 77 | Q27 | 1 | 3 | 16S V3-V4 |
| 78 | Q27B | 1 | 3 | 16S V3-V4 |
| 79 | Q27C | 1 | 3 | 16S V3-V4 |
| 80 | Q19 | 1 | 3 | 16S V3-V4 |
| 81 | Q19B | 1 | 3 | 16S V3-V4 |
| 82 | Q19C | 1 | 3 | 16S V3-V4 |
| 83 | Q18B | 1 | 3 | 16S V3-V4 |
| 84 | Q18C | 1 | 3 | 16S V3-V4 |
| 85 | Q21 | 1 | 3 | 16S V3-V4 |
| 86 | Q21B | 1 | 3 | 16S V3-V4 |
| M | D2000 DNA Marker | 1 | 3 |  |
|  | NTC | 1 | 3 | 16S V3-V4 |
|  | NTC | 1 | 3 | 16S V3-V4 |
| M | D2000 DNA Marker | 1 | 3 |  |
