# Supplementary material for: Influence of periodontal treatment on blood microbiotas: a clinical trial
Source: PeerJ. 2021 Feb 16;9:e10846. doi: 10.7717/peerj.10846 (PMC7894104; doi:10.7717/peerj.10846)
Supplement: Supplemental Information 10 [file peerj-09-10846-s010.pdf]

扩增子实验操作步骤

1. 基因组 DNA 提取

使用 PowerSoil DNA Isolation Kit(MoBio Laboratories, Carlsbad, CA)试剂盒提取基因组 DNA。

2. 基因组 DNA 质检

使用 Nanodrop 检验 DNA 质量和浓度。

3. PCR 扩增

按指定扩增区域，合成带有 barcode 的特异引物，或合成带有错位碱基的融合引物。使用 KAPA 2G Robust Hot Start Ready Mix 及相应引物进行 PCR 扩增（不同引物程序不同）。

PCR 扩增总体系为 25 μL：

试剂成分	体积
DNA 样品	X (30 ng)
Forward Primer (5 μM)	1 μL
Reverse Primer (5 μM)	1 μL
KAPA 2G Robust Hot Start Ready Mix	12.5 μL
ddH_2O	10.5-X μL
Total	25 μL

反应程序：

温度	反应时间	反应循环数
95℃	5 min	-
95℃	45 s	28 cycle
55℃	50 s	
72℃	45 s	
72℃	10 min	-
4℃	∞	-

4. PCR 产物电泳检测

1%琼脂糖凝胶电泳检测扩增目的条带大小，170 V 30 min。

5. PCR 产物纯化

PCR 产物使用磁珠法进行自动化纯化。

（1）磁珠室温平衡 30 min 后震荡混匀。取 18 μL 磁珠于新的 96 孔板中。

（2）将 PCR 产物加入装有磁珠的 96 孔板中混匀，室温静置 5 min。

（3）将 96 孔板置于磁力架上静置，使磁珠充分沉降，吸取并弃去上清。

（4）用 200 μL 80%乙醇吹打洗涤磁珠后，吸取并弃去酒精（此过程不要触碰到磁珠且不要将磁珠吹散，96 孔板不要离开磁力架）。

（5）继续用 200 μL 80%乙醇洗涤，吸取并弃去酒精，将含有磁珠的 96 孔板置于 37℃加热器上，烘干残余酒精。

（6）加入 27 μL EB solution，反复吹打混匀，室温静置 5 min，置于磁力架上澄清 2 min，回收 25 μL 于新的 96 孔板中。

（7）使用 Caliper 检测 PCR 产物浓度、片段大小。

6. Miseq 文库构建

按照 Pooling 比例，对没有错位碱基的融合引物 PCR 产物取一定体积 Pooling 成一个上机文库，并使用 2%琼脂糖凝胶对文库片段进行筛选。筛选后的文库片段经 Qubit 检测定量，取一定量文库加入 10 μL End repair & Add A 进行末端修复加 A 尾，再加入 33.5 μL Adaptor Ligation Mix 连接测序接头，并对文库进行纯化回收。然后加入接头引物、酶及 Mix 进行 PCR 富集完成文库构建。

PCR 富集反应程序：

温度	反应时间	反应循环数
37℃	15 min	-
98℃	30 s	5 cycle
98℃	10 s	
65℃	75 s	
65℃	5 min	-
4℃	∞	-

最后，构建完成的 Miseq 文库使用磁珠法进行纯化。

（1）磁珠室温平衡 30 min 后震荡混匀。取 40 μL 磁珠于新的 1.5 mL EP 管中。

（2）将 PCR 产物加入装有磁珠的 EP 管中，吹打混匀，室温静置 4 min（每个都不得超过 4 min，防止吸附引物二聚体）。

（3）将 EP 管置于磁力架上静置，使磁珠充分沉降，吸取并弃去上清。

（4）用 200 μL 80%乙醇吹打洗涤磁珠后，吸取并弃去酒精（此过程不要触碰到磁珠且不要将磁珠吹散，EP 管不要离开磁力架）。

（5）继续用 200 μL 80%乙醇洗涤 2 遍，将含有磁珠的 EP 管置于 37℃加热器上，烘干残余酒精。

（6）加入 27 μL EB solution，反复吹打混匀，室温静置 4 min，置于磁力架上澄清 2 min，回收 26 μL 于新的 EP 管中。

7. Miseq 文库质检

使用 Nanodrop 粗检文库浓度，Agilent 2100 检测文库片段，qPCR 精确定量文库浓度。

8. 上机测序

最后文库在 Illumina Miseq 平台上机测序。
