# Supplementary material for: Influence of periodontal treatment on blood microbiotas: a clinical trial
Source: PeerJ. 2021 Feb 16;9:e10846. doi: 10.7717/peerj.10846 (PMC7894104; doi:10.7717/peerj.10846)
Supplement: Supplemental Information 11 [file peerj-09-10846-s011.docx]

**1 DNA extraction**

Soil DNA was extracted using commercial kit (TianGen, TIANamp, China) following the manual. The concentration and purity of DNA were assessed with the Micro Drop Ultra Micro Spectrophotometer (Bio-DL, TX, USA). Double distilled water was used as the negative control during the extraction process.

**2 PCR amplification**

Nested polymerase chain reaction (nested PCR) amplification and 16S rRNA Gene Sequencing. Universal bacterial primers (27F 5’AGAGTTTGATCCTGGCTCAG-3’1492R5’-TACGGYTACCTTGTTACGACTT-3’) (Fredriksson et al., 2019) were used to detect bacterial DNA in the blood samples. PCR amplification was carried out in a 25 μl reaction mixture containing 2× Taq Plus Master Mix (KAPA 2G Robust HotStart ReadyMix, Sigma-Aldrich, USA), 5 μM of the forward and reverse primers, 6 ng bovine serum albumin (BSA), and 30 ng of template DNA. A 2 kb DNA ladder was used as a size marker to assist the analysis of the PCR products. Subsequently, nested PCR was performed using the universal primers (336F 5’-GTACTCCTACGGGAGGCAGCA-3’ 806R 5’-GTGGACTACHVGGGTWTCTAAT-3’) (Wei et al., 2016) targeting the V3-V4 region of the 16S rRNA gene. Doubled distilled water was also used as the negative control.

The final PCR products were separated by 2% agarose gel electrophoresis. The ~470 bp fragments obtained were purified using a QIAquick Gel Extraction Kit (Qiagen, Hilden, Germany)

**3 High throughput sequencing**

Deep sequencing was performed on Miseq platform at Allwegene Company (Beijing). After the run, image analysis, base calling and error estimation were performed using Illumina Analysis Pipeline Version 2.6.

**4 Data analyses**

The raw data were first screened and sequences were removed from consideration if they were shorter than 230 bp, had a low quality score (≤ 20), contained ambiguous bases or did not exactly match to primer sequences and barcode tags. Qualified reads were separated using the sample-specific barcode sequences and trimmed with Illumina Analysis Pipeline Version 2.6. And then the dataset were analyzed using QIIME. The sequences were clustereds into operational taxonomic units (OTUs) at a similarity level of 97%, to generate rarefaction curves and to calculate the richness and diversity indices. The Ribosomal Database Project (RDP) Classifier tool was used to classify all sequences into different taxonomic groups.

To examine the similarity between different samples, clustering analyses and PCA were used based on the OTU information from each sample using R. The evolution distances between microbial communities from each sample were calculated using the the Bray Curtis algorithms and represented as an Unweighted Pair Group Method with Arithmetic Mean (UPGMA) clustering tree describing the dissimilarity (1-similarity) between multiple samples. A Newick-formatted tree file was generated through this analysis. To compare the membership and structure of communities in different samples, heat maps were generated with the top 20 OTUs using Mothur.
